# The effects of hemodynamic management using the trend of the perfusion index and pulse pressure variation on tissue perfusion: a randomized pilot study

**DOI:** 10.1186/s40981-019-0291-5

**Published:** 2019-11-04

**Authors:** Kohei Godai, Akira Matsunaga, Yuichi Kanmura

**Affiliations:** 1 Department of Anesthesiology and Critical Care Medicine, Kagoshima University, Kagoshima, Japan; 2 Operating Room, Kagoshima University Hospital, Kagoshima, Japan

**Keywords:** Hemodynamic management, Perfusion index, Pulse pressure variation

## Abstract

**Background:**

Intraoperative hemodynamic management is challenging because precise assessment of the adequacy of the intravascular volume is difficult during surgery. Perfusion index (PI) has been shown to reflect changes in peripheral circulation perfusion. Pulse pressure variation (PPV) reflects the preload responsiveness. The hypothesis of this study was that hemodynamic management using the trend of the PI and PPV would improve tissue perfusion.

**Methods:**

This was a prospective, randomized, parallel design, single-blind, single-center pilot study. Patients undergoing elective open gynecological surgery requiring a direct arterial line were included. The patients were randomly allocated to two groups. The intervention group received hemodynamic management using the trend of the PI and PPV in an effort to improve tissue perfusion. The control group received hemodynamic management at the discretion of the anesthesia care provider. The primary outcome was the peak lactate level during surgery. The secondary outcomes were the duration of hypotension, intraoperative fluid balance, intraoperative urine output, and postoperative complication rate. Statistical analysis was performed using Student’s *t* test and Fisher’s exact test. A *P* value of < 0.05 was considered statistically significant.

**Results:**

Although the intervention significantly decreased the duration of hypotension and intraoperative fluid balance, the peak lactate level was not different between the intervention group and the control group. Intraoperative urine output and postoperative complication rate were not different between the groups.

**Conclusion:**

Hemodynamic management using the trend of the PI and PPV does not improve tissue perfusion in patients undergoing open gynecological surgery.

**Trial registration:**

This trial was prospectively registered on a publicly accessible database (UMIN Clinical Trials Registry ID: UMIN 000026957. Registered 12 April 2017, https://upload.umin.ac.jp/cgi-open-bin/ctr_e/ctr_view.cgi?recptno=R000030916).

## Background

Intraoperative hemodynamic management is challenging because precise assessment of the adequacy of the intravascular volume is difficult during surgery [1]. Although goal-directed fluid therapy (GDFT) has been shown to reduce perioperative complications in high-risk patients, some studies have shown conflicting results, i.e., GDFT did not reduce complications [2–4]. Challand et al. showed that stroke volume (SV) optimization using colloid bolus administration without vasopressors increased the length of hospital stay [3]. These studies have indicated that GDFT alone is insufficient for optimizing the hemodynamic status during surgery. To ensure adequate tissue perfusion, maintaining appropriate perfusion pressure is also crucial. Hypotension is reportedly associated with adverse perioperative outcomes [5–7].

The perfusion index (PI) has been shown to reflect changes in peripheral circulation perfusion [8]. Peripheral perfusion is influenced by cardiac output (CO) and peripheral vasomotor tone [9] and is sensitive to sympathetic vasoconstriction and skin temperature [10, 11]. The PI might be used as a surrogate of CO if sympathetic tone and skin temperature are kept stable. On the other hand, dynamic indices including pulse pressure variation (PPV) and stroke volume variation (SVV) have been used as indicators of fluid responsiveness. A PPV of > 13% during mechanical ventilation using a tidal volume of 8 mL/kg reliably predicts preload responsiveness [12]. A high PPV indicates that the patient’s left ventricle is positioned at the “steep” part of the Frank–Starling curve [13].

Although the SV and CO can be measured intraoperatively with several devices such as esophageal Doppler or pulse contour analysis, the cost of these devices may impede the widespread use of SV/CO measurement [14]. The anesthetic monitor Life Scope J (Nihon Kohden, Tokyo, Japan) provides PI and PPV measurements without any other devices. Anesthesiologists can evaluate the PI and PPV if a pulse oximeter and direct arterial line are placed in the patient. We developed a new hemodynamic protocol using the trend of the PI and PPV to maintain adequate tissue perfusion, which would improve perioperative outcomes without expensive SV/CO monitoring devices. The hypothesis of this study was that the new hemodynamic management using the trend of the PI and PPV would improve tissue perfusion.

## Methods

This manuscript adheres to the applicable CONSORT guidelines. The supporting CONSORT checklist is available as Additional file 1. This prospective, randomized, parallel design, single-blind, single-center clinical trial was approved by the ethics committee of Kagoshima University Hospital (#28-191) and was conducted from May 2017 to November 2018 at Kagoshima University Hospital. This trial was prospectively registered on a publicly accessible database (UMIN Clinical Trials Registry ID: UMIN 000026957. Registered 12 April 2017, https://upload.umin.ac.jp/cgi-open-bin/ctr_e/ctr_view.cgi?recptno=R000030916). Written informed consent was obtained from all participants.

The inclusion criteria were an American Society of Anesthesiologists physical status of I to III and performance of elective open gynecological surgery requiring a direct arterial line. The exclusion criteria were a history of uncompensated cardiac disease, stroke, arrhythmias, and severe liver/renal dysfunction.

### Protocol

Before the induction of general anesthesia, a thoracic or lumbar epidural catheter (17G Tuohy needle, Hakko disposable epidural catheter; Hakko, Nagano, Japan) was placed at T10 to L1 according to the incision level, and 3 mL of 1% mepivacaine without epinephrine was administered. General anesthesia was induced with propofol (1.0–2.0 mg/kg), remifentanil (0.3–0.5 μg/kg/min), and rocuronium (0.6–1.0 mg/kg). Anesthesia was maintained with desflurane (0.6–0.7 age-adjusted minimum alveolar concentration) and remifentanil (0.05–0.5 μg/kg/min) and intermittent bolus administration of rocuronium (10 mg) and fentanyl (2–10 μg/kg). The rate of remifentanil infusion was tailored to control hemodynamic responses. Rocuronium was used to maintain a train-of-four ratio of ≤ 1 (TOF-Watch SX; Organon, Dublin, Ireland). The Life Scope J was used to continuously monitor the heart rate, direct arterial blood pressure, electrocardiogram, peripheral oxygen saturation, end-tidal carbon dioxide tension (ETCO_2_), PPV, PI derived from the pulse oximeter plethysmographic waveform, bladder temperature, and skin temperature of the hand. The PPV is calculated from the arterial waveform using the following formula: PPV = [(maximum pulse pressure) − (minimum pulse pressure)]/[(maximum pulse pressure) + (minimum pulse pressure)] × 1/2 × 100 and reflects the preload responsiveness [13]. The PI is calculated from the infrared signal using the following formula: PI = (pulsatile signal/nonpulsatile signal) × 100. To assess the hemodynamic effects of the intervention, we continuously monitored the cardiac index (CI) and SV variation (SVV) with a high-fidelity dedicated pressure transducer (FloTrac sensor; Edwards Lifesciences, Irvine, CA, USA) and the Vigileo monitor, software version 3.01 (Edwards Lifesciences). The CI was calculated based on real-time analysis of the arterial waveform during a 20-s period. This calculation was performed at a sample rate of 100 Hz without the need for prior calibration using a proprietary algorithm based on the principle that the aortic pulse pressure is proportional to the SV.

**Fig. 1:**
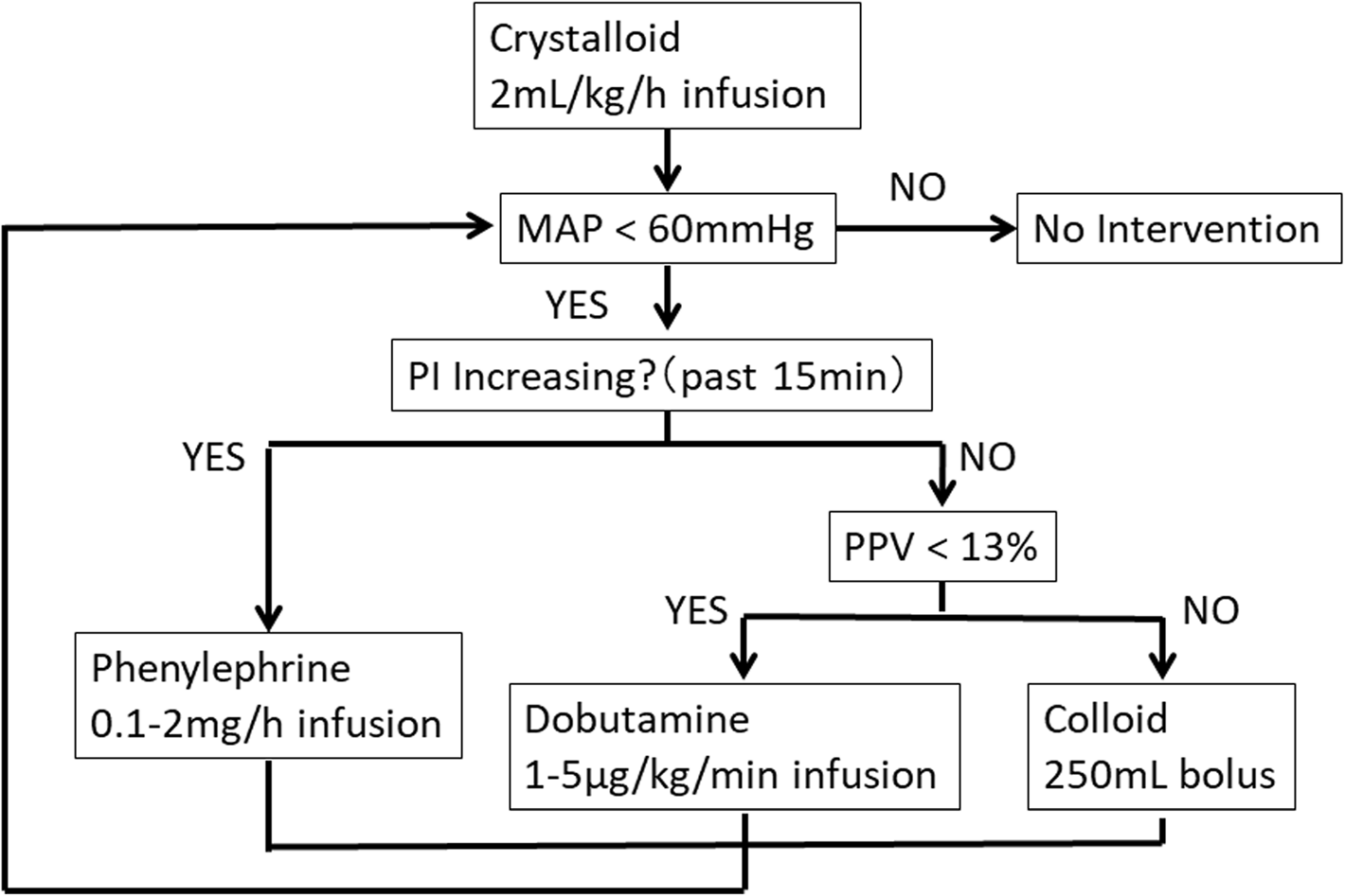
Hemodynamic management protocol using the trend of the perfusion index and pulse pressure variation. Abbreviations: MAP, mean arterial pressure; PI, perfusion index; PPV, pulse pressure variation

The patients’ lungs were ventilated with an inspired oxygen fraction of 0.4 and tidal volume of 8 mL/kg of ideal body weight, and the respiratory rate was adjusted to maintain an ETCO_2_ of 35 to 45 mmHg and a positive end-expiratory pressure of 5 to 8 cmH_2_O. The patients were randomly allocated to one of two groups with an allocation ratio of 1:1 using Internet-based software in a complete randomization manner (Research Randomizer version 4.0, retrieved on October 13, 2016, from http://www.randomizer.org/). The patients were blinded to the allocation. In the intervention group, 250 mL of colloid (Voluven; Otsuka Pharmaceutical, Tokyo, Japan) was infused during induction, followed by continuous infusion of a balanced crystalloid (Bicanate; Otsuka Pharmaceutical) at a rate of 2 mL/kg/h. In addition to the balanced crystalloid, 4.3% dextrose solution (Soldem 3A; Terumo, Tokyo, Japan) was administered at a rate of 20 mL/h. If the MAP was < 60 mmHg, the trend of the PI was evaluated (Fig. 1). We considered that 5% of change is clinically significant when we focused on the trend of the PI. If the PI increased by > 5% of previous value in 15 min, we considered that the hypotension was due to afterload reduction and administered a 0.1-mg bolus of phenylephrine followed by continuous infusion of phenylephrine at a rate of 1.0 mg/h. The infusion rate of phenylephrine was adjusted every 10 min (0.1–2.0 mg/h) to maintain the MAP within 60 to 90 mmHg. If the PI did not increase by > 5% of the previous value in 15 min, we evaluated the PPV. If the PPV was < 13%, we considered that the hypotension was due to reduced cardiac contractility and administered a continuous infusion of dobutamine at a rate of 3 μg/kg/min. The infusion rate of dobutamine was adjusted every 10 min (1–5 μg/kg/min) to maintain the MAP within 60 to 90 mmHg. If the PPV was ≥ 13%, we considered that the hypotension was due to preload reduction and administered a 250-mL bolus of colloid (Voluven; Otsuka Pharmaceutical). We stopped the infusion of phenylephrine or dobutamine when the MAP was above 90 mmHg with a minimum infusion rate. There is a consensus that MAP blow 60–70 mmHg is associated with perioperative complications [15]. We selected the threshold of 60 mmHg because we included the patients with low to moderate risk. In the control group, the hemodynamic management was performed at the discretion of the anesthesia care provider to maintain MAP more than 60 mmHg. These hemodynamic management were applied during surgery only. In both groups, red blood cells were transfused when the hemoglobin level was < 7 g/dL. Arterial blood samples were taken at the time of skin incision, every 2 h during surgery, and at the end of surgery. The lactate concentration was measured using an ABL 620 analyzer (Radiometer, Copenhagen, Denmark). Epidural analgesia was started at the closure of the skin. Postoperative complications were recorded during the first 30 days after surgery; these included pulmonary infection and infection of other organs as well as leakage at the anastomosis site.

### Data analysis

The primary outcome was the peak lactate level during surgery. The secondary outcomes were the duration of hypotension (MAP of < 60 mmHg), intraoperative fluid balance, intraoperative urine output, and postoperative complication rate. We considered 0.5 mmol/L difference in the lactate levels as clinically significant referred to the previous study [16]. To detect a 0.5 mmol/L difference in the peak lactate level with a two-sided approximation while accepting an α error of 5% and β error of 20%, the required study size was calculated as 34 patients based on the preliminary data using Power and Sample Size Calculation version 3.1.2 (Dupont WD and Plummer WD, Vanderbilt University, Nashville, TN, USA). The preliminary data were calculated from patients’ data who underwent open gynecological surgery in our institution (number of patients, 20; mean value of lactate levels, 1.2, standard deviation, 0.50). To account for patient dropout, 20% more patients were added, giving a final sample size of 40 patients. All data were expressed as mean with standard deviation or 95% confidence interval. The equality of variance was examined with an *F* test. The statistical analysis was performed using Student’s *t* test for data following Gaussian distribution, Mann-Whitney test for data not following Gaussian distribution, and Fisher’s exact test (GraphPad Prism 7.03; GraphPad Software, La Jolla, CA, USA). A *P* value of < 0.05 was considered statistically significant.

## Results

**Fig. 2:**
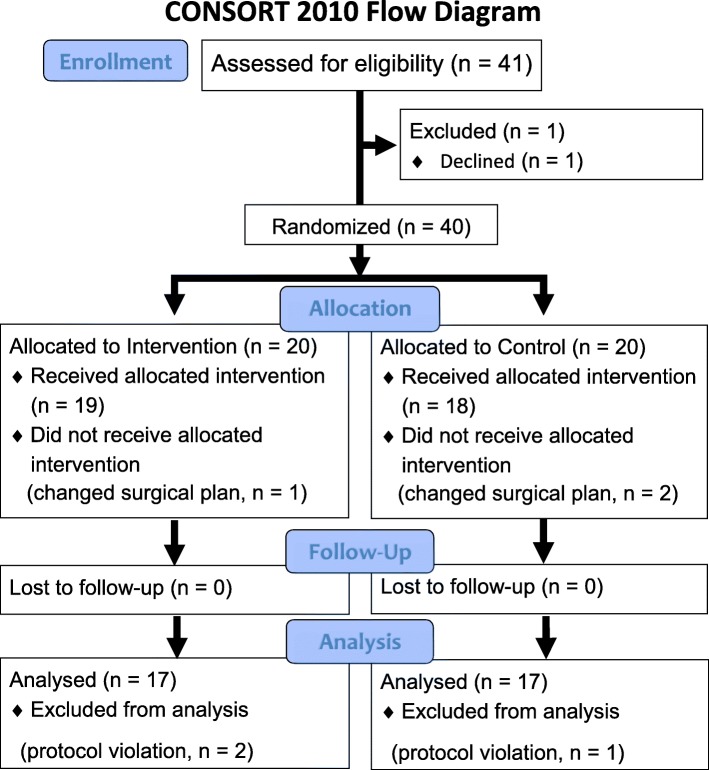
The CONSORT diagram

The CONSORT diagram is shown in Fig. 2. Of 41 patients considered eligible for the study, 1 patient declined to participate. Thus, 40 patients were included in the study. Three patients did not receive their allocated intervention because of a changed surgical plan and protocol violations. Another three patients’ data were not analyzed because of a protocol violation. Therefore, the data from 34 patients were analyzed. Table 1 shows the patients’ characteristics. Most patients underwent a radical hysterectomy. No patient had severe respiratory dysfunction, received preoperative β-blocker medications, or was transfused preoperatively.

**Table 1:** Patient characteristics

	Intervention group (*n* = 17)	Control group (*n* = 17)
Age (years)	49 ± 11	53 ± 17
Height (cm)	156 ± 6	157 ± 6
Weight (kg)	61 ± 13	55 ± 12
ASA-PS		
1	4 (24)	6 (35)
2	13 (76)	11 (65)
Comorbidities		
Obesity	3 (18)	1 (6)
Smoking	1 (6)	1 (6)
Hypertension	5 (29)	6 (35)
Anemia	5 (29)	3 (18)
Asthma	2 (12)	0 (0)
Diabetes mellitus	4 (24)	1 (6)
Types of surgery		
Radical hysterectomy	12 (71)	14 (82)
Radical trachelectomy	2 (12)	0 (0)
Salpingo-oophorectomy	3 (18)	3 (18)

*Abbreviation*: *ASA-PS* American Society of Anesthesiologists physical status

Values are expressed as mean ± standard deviation or number and percentage

### The primary and secondary outcomes

Although the intervention significantly decreased the duration of hypotension and intraoperative fluid balance, the peak lactate level was not different between the intervention group and the control group (Table 2). The number of patients whose peak lactate level was > 2.0 mmol/L was two in the intervention group and three in the control group (*P* > 0.99).

**Table 2:** Primary and secondary outcomes

	Intervention group (*n* = 17)	Control group (*n* = 17)	*P* value
Peak lactate levels (mmol/L)	1.4 ± 1.0	1.2 ± 0.5	0.98^b^
Duration of hypotension (min/h)	7.7 ± 5.0	17.1 ± 10.6	0.003^b^
Intraoperative fluid balance (mL/kg/h)	4.3 ± 1.3	7.2 ± 3.3	0.003^b^
Intraoperative urine output (mL/kg/h)	0.9 ± 0.8	1.2 ± 1.0	0.42^a^
Postoperative complication rate (%)	0	0	> 0.99^c^

Values are expressed as mean ± standard deviation or percentage

^a^*P* values were calculated using Student’s *t* test

^b^*P* values were calculated using Mann-Whitney test

^c^*P* values were calculated using Fisher’s exact test

### The other intraoperative data

Table 3 shows the intraoperative data. The MAP and SVV were significantly higher in the intervention group than in the control group. Patients in the intervention group were given less crystalloid intraoperatively than patients in the control group. Although the number of patients who received a vasopressor was not different between the groups, the dose of phenylephrine was higher in the intervention group. The number of patients whose mean PI was < 1.4% was five in the intervention group and three in the control group (*P* = 0.69). There was no difference in the respiratory parameters between the groups. There was no important harm in either group. Only one patient in the intervention group developed acute kidney injury (*P* > 0.99). The length of hospital stay did not differ in the groups (15 ± 8 days in the intervention group vs 14 ± 5 days in the control group, *P* = 0.67). No patient died within 30 days.

**Table 3:** Intraoperative data

	Intervention group (*n* = 17)	Control group (*n* = 17)	*P* value
Duration of anesthesia (min)	342 ± 96	332 ± 85	0.74^a^
Duration of surgery (min)	238 ± 89	239 ± 85	0.98^a^
Intraoperative blood loss (mL)	468 ± 466	485 ± 407	0.91^a^
HR (beats/min)	71 ± 11	75 ± 11	0.30^a^
MAP (mmHg)	74 ± 6	69 ± 7	0.04^a^
PI (%)	2.4 ± 1.6	2.8 ± 1.5	0.45^a^
PPV (%)	9.8 ± 3.8	8.8 ± 2.7	0.39^a^
SVV (%)	12.1 ± 3.8	9.6 ± 2.2	0.02^b^
CI (L/min/m^2^)	2.9 ± 0.7	3.3 ± 1.0	0.27^a^
SVI (mL/m^2^)	41 ± 11	42 ± 8	0.90^a^
Crystalloid (mL/kg/h)	3.8 ± 1.1	6.2 ± 2.1	0.0006^b^
Colloid (mL/kg/h)	2.3 ± 1.2	3.3 ± 3.0	0.61^b^
Number of patients transfused with RBC (*n*)	4	5	> 0.99^c^
RBC transfusion (mL)	135 ± 256	112 ± 203	0.77^a^
Number of patients received ephedrine (*n*)	15	13	0.66^c^
Total dose of ephedrine (mg)	9.3 ± 8.1	10.5 ± 8.4	0.31^a^
Ephedrine (mg/h)	1.5 ± 1.1	2.1 ± 1.7	0.68^a^
Number of patients received phenylephrine (*n*)	16	14	0.60^c^
Phenylephrine (mg/h)	0.73 ± 0.44	0.22 ± 0.32	0.0006^a^
Number of patients received dobutamine (*n*)	3	0	0.23^c^
Dobutamine (μg/kg/min)	0.14 ± 0.49	0.00 ± 0.00	0.23^b^
ETCO_2_ (mmHg)	37 ± 1.4	37 ± 1.4	0.39^a^
TV (mL/kg of IBW)	8.2 ± 0.4	8.3 ± 0.8	0.84^b^
Ppeak (cmH_2_O)	18 ± 2.7	17 ± 1.8	0.07^a^
PEEP (cmH_2_O)	5.1 ± 0.86	4.7 ± 0.7	0.11^a^
ETDes (%)	3.7 ± 0.3	3.5 ± 0.5	0.31^b^
Tblad (°C)	36.9 ± 0.6	36.6 ± 0.6	0.18^a^
Tskin (°C)	36.1 ± 0.5	35.9 ± 1.1	0.95^b^

*Abbreviations*: *CI* cardiac index, *ETCO*_*2*_ end-tidal carbon dioxide tension, *ETDes* end-tidal desflurane concentration, *HR* heart rate, *IBW* ideal body weight, *MAP* mean arterial pressure, *PEEP* positive end-expiratory pressure, *PI* perfusion index, *Ppeak* peak inspiratory pressure, *PPV* pulse pressure variation, *RBC* red blood cell, *SVI* stroke volume index, *SVV* stroke volume variation, *Tblad* bladder temperature, *Tskin* skin temperature of the hand, *TV* tidal volume

Values are expressed as mean ± standard deviation or number

^a^*P* values were calculated using Student’s *t* test

^b^*P* values were calculated using Mann-Whitney test

^c^*P* values were calculated using Fisher’s exact test

## Discussion

Hemodynamic management using the trend of the PI and PPV did not decrease the intraoperative peak lactate level. Among the secondary outcomes, the intervention significantly decreased the duration of hypotension and intraoperative fluid balance. The MAP and SVV were higher in the intervention group than the control group, although less crystalloid was infused in the intervention group.

Our protocol was unique at two points. First, we avoided using SV or CO. Second, our protocol enables us to determine which factor is responsible for hypotension only using PI and PPV. We selected open gynecological surgery to reduce variability. Because the PI is sensitive to sympathetic vasoconstriction and skin temperature, we maintained normothermia [10]. A PI of < 1.4% has been reported to represent poor peripheral perfusion in clinically ill patients [8]. The number of patients whose mean PI was < 1.4% was not different between the groups. Because the PI reportedly has considerable inter-individual variability, we used the trend of the PI.

Our hypothesis that hemodynamic management using the trend of the PI and PPV would improve tissue perfusion was not confirmed in this study. There are several possible explanations for why our hypothesis failed to be confirmed. One simple explanation is that hemodynamic management did not decrease the intraoperative lactate level because the intervention did not improve tissue perfusion. Another explanation is that the risk of tissue hypoperfusion might be too low to detect the difference. The previous study, however, has reported that GDFT using the pulse oximeter–derived pleth variability index reduced lactate levels in the moderate risk patients [16].

We selected the single cutoff point of PPV as 13% because we needed to make a simple protocol. As Cannesson et.al. have shown, the gay zone approach is another option to make a clinical decision [17]. We have shown that phenylephrine infusion significantly decreased the PPV during one-lung ventilation [18]. In this study, although the doses of phenylephrine were higher in the intervention group than in the control group, PPV was not different between the groups. The differences between the results of our previous study and the current study may be due to the concomitant colloid bolus or dobutamine infusion. No serious adverse events occurred during this study.

Our study has several limitations. First, we included a moderate to low risk female patients only. Our results cannot be generalized to high risk or male patients. Second, we used the peak lactate level as a surrogate of tissue perfusion. Because we did not measure tissue perfusion directly using ultrasound or near-infrared spectroscopy, the actual tissue perfusion cannot be assessed. Third, we did not use any particular hemodynamic protocols in the control group. It is possible that the results might be changed if we used particular hemodynamic protocols which may reduce variability in the control group.

## Conclusion

Although hemodynamic management using the trend of the PI and PPV significantly decreased the duration of hypotension and intraoperative fluid balance, the intervention did not decrease the intraoperative peak lactate level. These results indicate that hemodynamic management using the trend of the PI and PPV does not improve tissue perfusion in patients undergoing open gynecological surgery.

## Supplementary information

**Additional file 1.** CONSORT 2010 checklist of information to include when reporting a randomised trial*.

## Data Availability

The datasets generated and analyzed during the current study are available in the figshare repository [19], https://figshare.com/articles/20190512_PI_PPV_study_data_repository/8115971.

## Abbreviations

CI: Cardiac index
CO: Cardiac output
ETCO_2_: End-tidal carbon dioxide tension
GDFT: Goal-directed fluid therapy
MAP: Mean arterial pressure
PI: Perfusion index
PPV: Pulse pressure variation
SV: Stroke volume
SVV: Stroke volume variation
